# Acute Sulfite-Induced Hypersensitivity Reaction Following Balsamic Vinegar Consumption: A Case Report

**DOI:** 10.7759/cureus.107225

**Published:** 2026-04-17

**Authors:** Akhtar Purvez, Ana Mir, Mudhasir Bashir

**Affiliations:** 1 Clinical Research, Momentum Medical Research, Charlottesville, USA; 2 Clinical Sciences, Lincoln Memorial University-DeBusk College of Osteopathic Medicine, Harrogate, USA; 3 Clinical Sciences, Momentum Medical Research, Charlottesville, USA; 4 Psychiatry and Behavioral Sciences, The University of Virginia, Charlottesville, USA

**Keywords:** autonomic reaction, balsamic vinegar, food additives intolerance, food preservative hypersensitivity, sulfite allergy

## Abstract

Sulfite sensitivity is an underrecognized cause of acute adverse reactions to preserved foods and beverages. Although sulfites are widely used as antimicrobial and antioxidant agents, hypersensitivity reactions may occur even in individuals without a prior allergy history. We report the case of a 68-year-old otherwise healthy male who developed an acute systemic reaction shortly after ingesting balsamic vinegar containing sulfites. The episode was characterized by severe epigastric burning pain, flushing, diaphoresis, and intense autonomic discomfort associated with a subjective sensation of heat requiring removal of clothing for relief. Symptoms resolved spontaneously within approximately one hour without medical intervention. The patient had long tolerated balsamic vinegar of Modena from another manufacturer without labeled sulfites and without adverse effects. This presentation suggests a sulfite-induced hypersensitivity reaction with predominant gastrointestinal and autonomic manifestations rather than classic respiratory findings typically associated with immunoglobulin E (IgE)-mediated reactions. Recognition of such reactions is important to prevent misdiagnosis and unnecessary diagnostic testing.

## Introduction

Sulfites are widely used food preservatives that serve as antioxidants and antimicrobial agents, helping to prevent oxidation, discoloration, and microbial spoilage in processed foods and beverages. Their incorporation into commercially prepared products has become routine in modern food production, particularly in wines, dried fruits, pickled foods, condiments, and vinegars, including balsamic vinegar preparations. As a result, repeated dietary exposure to sulfites is common in the general population. While regulatory labeling requirements aim to alert consumers to their presence, awareness of potential adverse reactions remains limited. Reported reactions to sulfites range from mild cutaneous flushing and gastrointestinal discomfort to bronchospasm, hypotension, and anaphylactoid events in susceptible individuals [1-3].

Sulfites are commonly used preservatives in foods and beverages, including balsamic vinegar, where they help prevent oxidation and maintain product stability. Although the overall prevalence of clinically significant sulfite sensitivity is low, reactions may occur in both asthmatic and non-asthmatic individuals, with variable clinical presentations.

Historically, sulfite sensitivity has been most strongly associated with asthma, especially in patients with more severe or corticosteroid-dependent disease. However, growing clinical recognition suggests that adverse reactions may also occur in individuals without underlying respiratory pathology [2,3]. In such cases, presentations may be atypical, manifesting predominantly through autonomic and gastrointestinal pathways rather than classic immunoglobulin E (IgE)-mediated allergic mechanisms. These atypical presentations may mimic acute coronary syndromes, gastroesophageal disorders, or panic-related phenomena, leading to diagnostic uncertainty and potential misattribution. Increased clinical vigilance and careful dietary history taking are therefore essential when evaluating acute symptoms temporally associated with food ingestion. This report describes a striking autonomic and gastrointestinal reaction occurring shortly after consumption of balsamic vinegar explicitly labeled as containing sulfites, underscoring the importance of preservative awareness and individualized susceptibility in contemporary clinical practice.

## Case presentation

A 68-year-old male with no significant past medical history and no regular medication use was evaluated in an outpatient clinical setting in Charlottesville, Virginia, following a self-limited episode of acute systemic discomfort that occurred shortly after consuming a small quantity, approximately one tablespoon, of balsamic vinegar during a meal.

**Figure 1:**
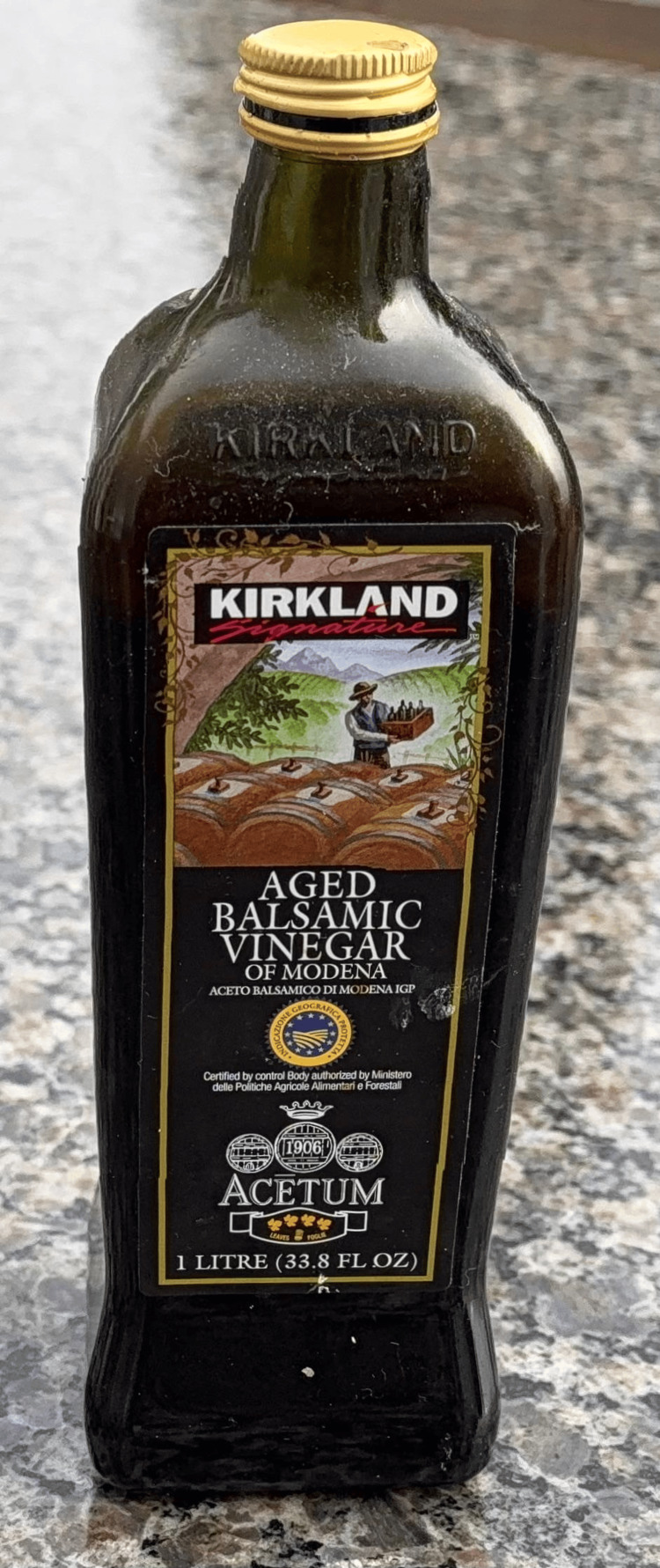
Full product image of the balsamic vinegar consumed by the patient.

The patient stated that he was in his normal state of health before the event and had not been sick, changed medications, drunk alcohol, or eaten any new foods apart from the specific balsamic vinegar product he had at that time (Figure 1).

He had a long-standing history of regularly consuming the balsamic vinegar of Modena from another manufacturer without any prior adverse reactions.

He experienced severe epigastric burning pain within minutes of ingestion, along with significant flushing, excessive sweating, and a strong feeling of internal heat. The discomfort was sufficiently severe that he removed his clothing in an attempt to obtain relief and cooling. He described a feeling of generalized internal agitation and autonomic distress but denied chest pressure typical of cardiac ischemia, palpitations, dyspnea, throat tightness, dysphagia, urticaria, angioedema, wheezing, lightheadedness, or syncope. No focal neurologic symptoms were reported.

Symptoms quickly reached their highest level of intensity, stayed that way for about an hour, and then slowly went away on their own without any medical treatment or medication. The patient avoided antihistamines and other medications, and emergency medical services were unnecessary. He did not seek hospital evaluation, as symptoms resolved completely within one hour, and no delayed or biphasic symptoms occurred during subsequent observation. No vital signs or physical examination findings were recorded, as the patient did not seek medical evaluation during the episode. Because the symptoms went away quickly and completely on their own, there was no need for any lab tests or heart tests.

The absence of laboratory evaluation reflects the self-limited nature of the episode, which resolved completely within one hour without residual symptoms, a pattern commonly observed in transient exposure-related reactions. No alternative dietary triggers or environmental exposures were identified. Table 1 gives a summary of the lab tests.

**Table 1:** Clinical evaluation and diagnostic considerations.

Category	Findings
Initial clinical context	Self-limited episode occurring immediately after food ingestion
Respiratory symptoms	No dyspnea, wheezing, or airway compromise
Allergic features	No urticaria, angioedema, or throat swelling
Neurologic symptoms	No focal deficits or altered consciousness
Clinical course	Rapid onset and spontaneous resolution within one hour
Laboratory evaluation	Not performed due to complete symptom resolution
Overall assessment	Findings did not suggest the need for emergent evaluation or laboratory testing

**Figure 2:**
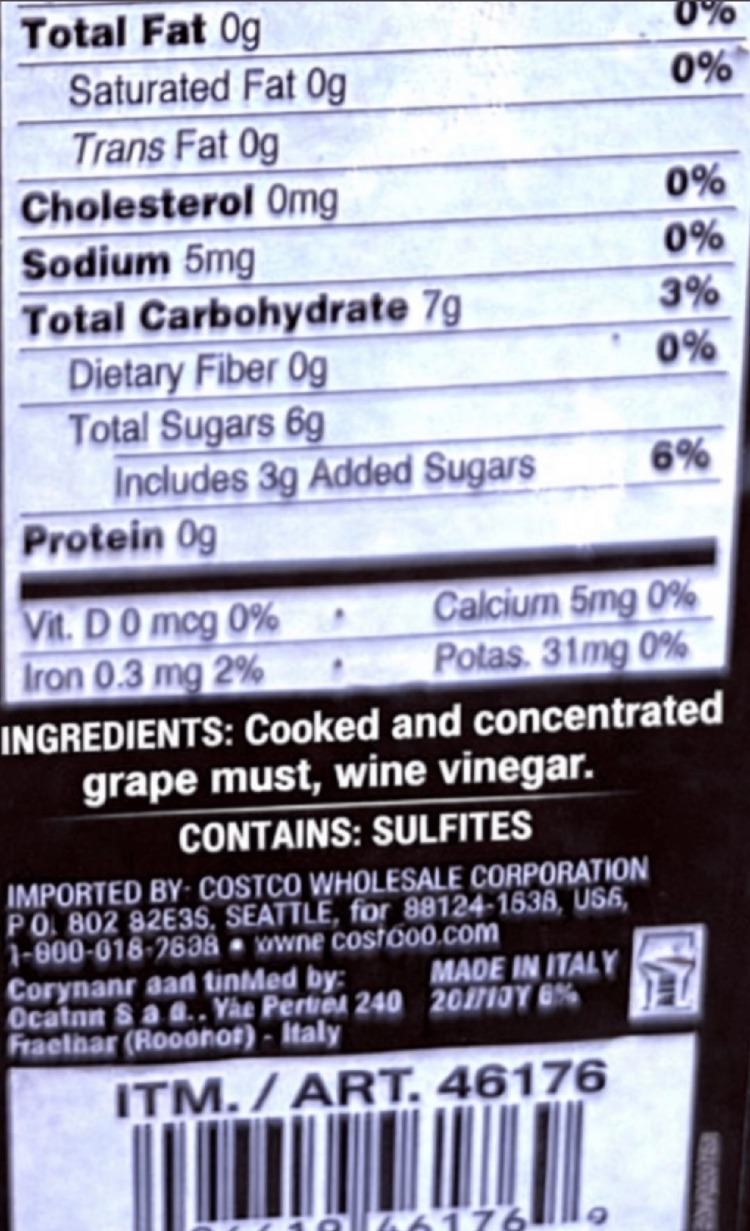
Ingredients list demonstrating the statement “CONTAINS: Sulfites.” Original product label of the specific balsamic vinegar consumed by the 68-year-old patient.

A review of the product label confirmed that the consumed balsamic vinegar explicitly listed sulfites among its contents (Figure 2).

Based on the temporal association, documented sulfite exposure, prior tolerance to sulfite-free products, and spontaneous symptom resolution, the presentation was considered most consistent with a probable sulfite-related hypersensitivity reaction; however, definitive confirmation was not established due to the absence of objective testing or rechallenge.

## Discussion

**Figure 3:**
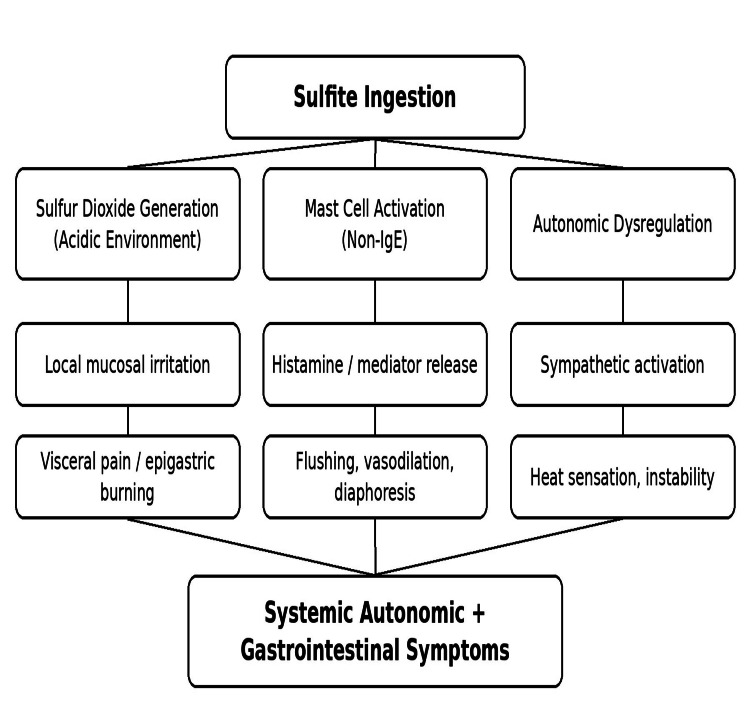
Proposed non-IgE-mediated mechanisms of sulfite reactions, including sulfur dioxide generation in acidic environments, mast cell activation, and autonomic dysregulation pathways. The patient’s presentation is most consistent with autonomic and gastrointestinal pathways illustrated in this diagram. IgE: immunoglobulin E Source: Image created using Microsoft PowerPoint (Microsoft Corporation, Redmond, WA, USA).

Sulfite reactions have been recognized for decades and may occur through several biological mechanisms, including non-IgE-mediated mast cell activation, sulfur dioxide generation in acidic environments, and impaired sulfite metabolism in susceptible individuals [4]. These mechanisms may contribute to variability in clinical presentation and symptom severity. The proposed mechanisms underlying sulfite-associated reactions are illustrated in Figure 3.

A broader clinical framework relevant to this case is the growing recognition of individualized physiologic responses to dietary and pharmacologic exposures. Prior reports have described acute systemic reactions following both pharmacologic agents and dietary triggers, including arrhythmogenic, renal, neurologic, and autonomic manifestations [5-11]. These observations suggest that susceptible individuals may exhibit exaggerated physiologic responses to otherwise well-tolerated exposures. While the underlying mechanisms remain incompletely understood, proposed pathways include autonomic dysregulation, mast cell activation, and metabolic variability, which may contribute to the heterogeneity of clinical presentations.

In the context of sulfite exposure, toxicologic and clinical observations demonstrate that sulfites may produce a spectrum of adverse effects depending on host susceptibility and exposure conditions [12]. Epidemiologic data suggest increased sensitivity among individuals with asthma, although reactions are not limited to this population [13]. More broadly, studies of food additive intolerance, including monosodium glutamate (MSG) and related compounds, support the concept that non-allergic chemical sensitivities can produce reproducible systemic symptoms that are best managed through recognition and avoidance of the triggering agent [14-16].

This case highlights the clinical relevance of sulfite hypersensitivity by demonstrating differential tolerance to similar products from different manufacturers. The patient’s long-standing tolerance of sulfite-free balsamic vinegar, contrasted with an acute reaction following ingestion of a sulfite-containing product, supports an exposure-related association.

Sulfite reactions may present with autonomic symptoms such as flushing, diaphoresis, and visceral burning, potentially mimicking conditions including acute coronary syndrome, gastritis, or panic disorder. Without careful dietary assessment, such presentations may lead to unnecessary diagnostic evaluation. Recognition of potential food preservative triggers and review of product labeling are therefore essential.

This episode is most consistent with a "probable" adverse reaction based on the World Health Organization-Uppsala Monitoring Centre (WHO-UMC) criteria, including a clear temporal relationship, absence of alternative explanations, and positive dechallenge. However, this classification reflects clinical probability rather than definitive causation, as objective diagnostic confirmation and rechallenge were not performed.

Table 2 summarizes the causality assessment, and Table 3 delineates the symptom patterns, observations, and clinical implications derived from this case. It is important to emphasize that the conclusions drawn in this report are based on clinical assessment and temporal association, and should be interpreted within the limitations of a single observational case without confirmatory testing.

**Table 2:** WHO-UMC causality assessment supporting a probable sulfite‑induced hypersensitivity reaction. WHO-UMC: World Health Organization-Uppsala Monitoring Centre

Temporal relationship	Present (symptoms within minutes of ingestion)
Dechallenge	Positive (spontaneous resolution after avoidance)
Rechallenge	Not performed
Alternative causes	Unlikely based on clinical history
Objective evidence	The label confirms the presence of sulfites
Prior tolerance of sulfite‑free products	Yes
WHO-UMC category	Probable adverse reaction

**Table 3:** Symptom patterns, observations, and clinical implications

Clinical domain	Key observation	Clinical implication
Symptom pattern	Sulfite hypersensitivity may present predominantly with gastrointestinal and autonomic symptoms rather than respiratory or dermatologic findings.	Clinicians should not rely solely on classic allergic manifestations when evaluating suspected food reactions.
Acute presentation	Acute flushing, diaphoresis, and severe epigastric burning following ingestion of preserved foods.	Temporal association with food exposure should prompt consideration of sulfite sensitivity.
Diagnostic mimics	Symptoms may resemble cardiac ischemia, panic disorder, or gastritis.	Misdiagnosis may lead to unnecessary diagnostic testing and delayed recognition of the trigger.
Preventive strategy	Careful dietary history and review of food labeling.	Identification and avoidance of sulfite-containing foods can prevent recurrent episodes.

Limitations

This case has several limitations. A formal rechallenge was not performed due to safety considerations, which limits definitive causality confirmation. No skin prick or oral challenge testing was conducted to exclude rare alternative hypersensitivity mechanisms. Laboratory evaluation was not conducted due to the swift and total spontaneous resolution of symptoms. Additionally, the diagnosis is based on clinical assessment and temporal association rather than objective biomarker confirmation. Despite these limitations, the strong temporal relationship, documented sulfite exposure, and prior tolerance of sulfite-free products support a clinically plausible exposure-related association.

## Conclusions

This case suggests that sulfite-containing foods may provoke clinically significant hypersensitivity-like reactions in susceptible individuals, even in the absence of underlying respiratory disease. The predominantly gastrointestinal and autonomic presentation observed here, supported by within-patient comparator evidence of long-standing tolerance to sulfite-free products, highlights the potential for atypical symptom patterns that may mimic cardiac, gastrointestinal, or anxiety-related conditions. Recognition of such presentations may help avoid unnecessary diagnostic evaluations and facilitate earlier identification of potential dietary triggers. This case also underscores the broader concept of food additive sensitivity, as certain individuals may exhibit exaggerated physiologic responses to commonly used additives such as sulfites and MSG. These observations should be interpreted cautiously, as they are derived from a single clinical case without confirmatory testing; however, increased awareness may improve diagnostic accuracy and support preventive strategies through targeted dietary modification and patient education.
